# Assessment of Direct Oral Anticoagulant Plasma Concentrations Before Elective Electrical Cardioversion

**DOI:** 10.3390/medicina62102007

**Published:** 2026-10-16

**Authors:** Anastasija Ņikitina, Inga Urtāne, Daniels Siļčonoks, Jānis Meisters, Gustavs Latkovskis, Anna Gavrilova

**Affiliations:** 1 Department of Pharmacology and Pharmacotherapy, Faculty of Pharmacy, Rīga Stradiņš University, LV-1007 Riga, Latviainga.urtane@rsu.lv (I.U.);; 2 Joint Laboratory, Pauls Stradiņš Clinical University Hospital, LV-1002 Riga, Latvia; 3 Institute of Cardiology and Regenerative Medicine, University of Latvia, LV-1586 Riga, Latvia; gustavs.latkovskis@lu.lv; 4 Latvian Center of Cardiology, Pauls Stradiņš Clinical University Hospital, LV-1002 Riga, Latvia

**Keywords:** atrial fibrillation, edoxaban, rivaroxaban, dabigatran, drug–drug interactions, amiodarone

## Abstract

*Background and Objectives*: Electrical cardioversion (ECV) restores sinus rhythm in patients with atrial fibrillation (AF) and requires uninterrupted anticoagulation to prevent thromboembolic events. Despite the predictable pharmacokinetic profile of direct oral anticoagulants (DOACs), variability in plasma concentrations and possible drug–drug interactions may have clinical implications. Therefore, this study aimed to describe DOAC plasma concentrations before elective ECV under routine clinical, using expected concentration ranges reported in the guidelines as descriptive reference points. *Materials and Methods*: A cross-sectional study included adult patients with AF receiving rivaroxaban, dabigatran, or edoxaban before elective ECV. Blood samples were collected before the procedure irrespective of the timing of DOAC administration, and plasma concentrations were measured using drug-specific chromogenic assays and compared with the expected peak and trough concentration ranges. Comparisons with expected peak and trough concentration distributions were descriptive only. *Results*: Thirty-seven patients were analysed, with polypharmacy observed in 83.8% of participants. Ten measurements (27.0%) exceeded the upper bounds of expected peak concentration distributions, while two measurements (5.4%) fell below the lower bounds of trough distributions. Two patients were receiving standard DOAC doses despite meeting renal criteria for dose reduction. *Conclusions*: Considerable variability in randomly timed DOAC concentrations was observed before ECV. Observed dosing discrepancies highlight the importance of periodically reassessing prescribed doses against current patient characteristics. Larger prospective studies with standardized sampling and clinical follow-up are needed to clarify the clinical significance of these findings.

## 1. Introduction

Atrial fibrillation (AF) is the most common form of cardiac arrhythmia encountered in clinical practice. Over recent decades, the estimated lifetime risk of AF has risen from around 1 in 5 to up to 1 in 3 individuals, a trend that largely reflects population ageing as well as improvements in diagnostic detection. AF has also been linked to increased epicardial adipose tissue volume and markers of coronary inflammation [1]. In response to this growing burden, the European Society of Cardiology (ESC) AF-CARE pathway provides a structured framework for management, emphasizing four core principles: systematic management of cardiovascular risk factors, prevention of thromboembolic events through appropriate anticoagulation, effective control of AF-related symptoms, and ongoing, patient-centred reassessment over time [2,3].

To translate these principles into clinical practice, both non-invasive and invasive treatment strategies are employed in the management of AF. Non-invasive approaches primarily include pharmacological therapy and electrical cardioversion (ECV), whereas invasive strategies mainly comprise catheter ablation techniques. ECV is an effective method for restoring sinus rhythm in patients with paroxysmal or persistent AF [3]. In routine clinical practice, ECV is used more frequently than catheter ablation, largely due to its lower technical complexity, shorter procedural duration, and broader availability across healthcare settings. In contrast, catheter ablation requires specialized electrophysiological expertise and infrastructure and is therefore predominantly performed in tertiary or specialized centers [4]. Consequently, ECV currently represents the most utilized strategy for rhythm restoration, playing a central role in everyday clinical management of AF [5]. To enhance the effectiveness of ECV and to reduce the risk of early arrhythmia recurrence, antiarrhythmic drug therapy is commonly initiated prior to the procedure, most frequently with amiodarone or class Ic antiarrhythmic agents [6].

In contemporary clinical practice, direct oral anticoagulants (DOACs), such as apixaban, rivaroxaban, dabigatran, and edoxaban, are increasingly used and have largely replaced vitamin K antagonists due to their more favourable safety profile and greater convenience of use [2]. This global trend is also reflected at the national level. According to data from the Latvian State Agency of Medicines, the defined daily doses of DOACs per 1000 inhabitants per day increased from 13.3 to 22.3 during the period from 2021 to 2025 [7]. This increasing utilization reflects the improved clinical profile of DOACs, which has established them as the preferred anticoagulant option across a broad range of patients.

Despite their advantageous clinical profile, the use of DOACs is associated with an increased risk of bleeding. Therefore, in most invasive procedures, anticoagulant therapy is temporarily adjusted. ECV, however, represents an exception, as the risk of thromboembolic events in this setting is particularly high. Following restoration of sinus rhythm, a transient impairment of atrial mechanical function, commonly referred to as atrial stunning, occurs, which facilitates thrombus mobilization, causing serious complications, especially ischemic stroke and systemic thromboembolism. Consequently, uninterrupted anticoagulation both before and after cardioversion is considered mandatory and constitutes a standard component of care in accordance with the recommendations of the ESC [3,6].

Within this therapeutic context, the primary objective of pharmacological therapy is to achieve an optimal balance between safety and efficacy; however, evidence indicates that the risk of bleeding increases in patients with elevated plasma concentrations of DOACs [8]. Conversely, low DOAC plasma concentrations may reduce anticoagulant efficacy and increase the risk of thromboembolic events [9].

Importantly, the concentration of the active substance is not fixed and may be influenced by several patient- and treatment-related factors. Among these, concomitant use of medications that act as inducers or inhibitors of enzymes involved in DOAC metabolism represents a clinically relevant determinant. DOAC concentrations may be affected by interactions involving P-glycoprotein-mediated transport and, for some DOACs, cytochrome P450-mediated metabolism [10,11]. In patients with structural heart disease or persistent arrhythmia, antiarrhythmic therapy with amiodarone is often required, and by inhibiting CYP3A4 and P-gp, amiodarone may increase DOAC exposure and bleeding risk [12].

Given the importance of anticoagulation before elective ECV and the frequent use of concomitant medications, this study aimed to describe DOAC plasma concentrations encountered during routine pre-cardioversion assessment, using expected concentration ranges reported in the guidelines as descriptive reference points for these real-world measurements [13].

## 2. Materials and Methods

This cross-sectional study was conducted at Pauls Stradiņš Clinical University Hospital (PSCUH) between August and December 2022. Ethical approval for this study was granted by the Central Medical Ethics Committee of Latvia (decision No. 10900, with amendments No. 5348, No. 5159, and No. 5373) and the research was carried out in accordance with the ethical standards of the Declaration of Helsinki. All patients were included in the study after providing written informed consent by signing the consent form.

The study included ambulatory patients whose visit to PSCUH was scheduled for ECV. Inclusion criteria were as follows: (1) age ≥ 18 years, (2) a diagnosis of AF, and (3) administration of a DOAC (rivaroxaban, dabigatran, or edoxaban) within 24 h prior to blood sampling. In this study, patients receiving apixaban were excluded due to the small number of apixaban users in Latvia during the study period. Exclusion criteria included: (1) cognitive impairment or dementia, (2) severe comorbidities likely to affect clinical prognosis (e.g., progressive malignant disease, stage IV chronic heart failure, or HIV/AIDS), and (3) inability to obtain sufficient blood volume for laboratory analysis.

Data were collected using a structured survey that captured demographic and clinical characteristics as well as medication use. Demographic variables included sex, age, body weight and height. Participants provided detailed information on all medications used, including prescription and over-the-counter drugs, as well as dietary supplements, and reported relevant medical history. Survey data were supplemented with information extracted from patients’ medical records and other available medical documentation, including diagnoses, prescribed pharmacotherapy, and clinical characteristics relevant to anticoagulant use. All reported medicinal substances were classified according to the Anatomical Therapeutic Chemical classification system. Polypharmacy was defined as the concurrent use of five or more prescription and over-the-counter active substances, excluding dietary supplements. Assessment of complications was limited to the hospital visit for ECV, including the procedure and the period before discharge. No post-discharge follow-up was conducted.

The appropriateness of each patient’s prescribed DOAC dose was assessed according to the relevant Summary of Product Characteristics and ESC guidance, considering Cockcroft–Gault creatinine clearance, age, body weight, and other applicable dose-reduction criteria, including concomitant verapamil for dabigatran and ciclosporin, dronedarone, erythromycin, or ketoconazole for edoxaban [3,14,15,16].

Blood samples were obtained before elective ECV irrespective of the timing of the preceding DOAC dose. The exact interval between the last DOAC dose and blood sampling was not systematically recorded. Adherence to DOAC therapy during the preceding at least three weeks was assessed through patient interviews and relied on self-reported information. All participants were fasting before ECV. Investigators were not authorized to alter routine departmental workflow, patients’ prescribed medication schedules, or their preparation for cardioversion. Patients were therefore not instructed to change or withhold DOAC doses or to attend for sampling at a predefined time relative to administration. This approach allowed chromogenic DOAC assays to be performed alongside standard pre-ECV laboratory testing and provided randomly timed concentration measurements under routine clinical conditions.

One sample was collected from each study participant using a 2.7 mL BD Vacutainer^®^ tube (Belliver Industrial Estate, Plymouth, UK) containing 3.2% sodium citrate. After collection, samples were transported to the laboratory at room temperature within four hours. Chromogenic assays were performed upon sample receipt to quantify DOAC concentrations. Rivaroxaban and edoxaban levels were measured using the BIOPHEN^®^ DiXaI assay (Hyphen Biomed, Neuville-sur-Oise, France) [17], while dabigatran levels were measured using the INNOVANCE^®^ DTI assay (Siemens Healthineers, Erlangen, Germany) [18].

The calibration ranges were 5–500 ng/mL for rivaroxaban and edoxaban and 20–500 ng/mL for dabigatran. Laboratory verification yielded coefficients of variation at low and high concentrations of 9.0% and 7.1% for rivaroxaban, 4.9% and 2.7% for edoxaban, and 9.1% and 4.1% for dabigatran, respectively.

Published peak and trough concentration ranges from the 2021 EHRA Practical Guide are presented in Table 1 for descriptive context [13].

The sample size was determined by the number of eligible patients recruited during the study period and the resources available for laboratory testing. No formal a priori sample-size calculation was performed. Given the small overall sample and dose-specific subgroups, the analysis was restricted to descriptive summaries rather than hypothesis testing or estimation of drug-specific associations.

The distribution of continuous variables was assessed using the Shapiro–Wilk test and visual inspection of Q–Q plots. Descriptive statistics were presented according to data distribution: normally distributed variables were summarised as arithmetic mean and standard deviation (±SD), whereas non-normally distributed variables were reported as median with the first and third quartiles [Q1–Q3]. Categorical variables were expressed as absolute numbers and percentages. Missing values were not imputed. Descriptive summaries were based on available observations. Statistical analyses were performed using IBM SPSS Statistics (version 29.0).

## 3. Results

### 3.1. Participant Characteristics

The study included 37 patients, and the mean duration of AF in the study population was 4.5 ± 4.8 years. No complications were recorded during ECV or in the period before discharge. The most prevalent comorbidities were arterial hypertension (62.2%), chronic heart failure (59.5%), and dyslipidemia (56.8%). A detailed overview of participants’ demographic and clinical characteristics is presented in Table 2.

On average, each study participant was prescribed 7.2 ± 2.6 medications. Two patients (5.4%) used at least one over-the-counter medication (with a single active ingredient), and nine (24.3%) used at least one dietary supplement. Polypharmacy (≥5 active medication substances, including DOAC) was observed in 83.8% of patients. Excluding DOACs, the most commonly used medications were beta-blockers (86.5%), statins (73.0%), and angiotensin-converting enzyme inhibitors (54.1%).

### 3.2. DOAC Concentration Values

Among the 37 participants, 18 received rivaroxaban, six received dabigatran, and 13 received edoxaban. Median randomly timed plasma concentrations [Q1–Q3] were 176 [104–246] ng/mL for rivaroxaban, 133 [99–179] ng/mL for dabigatran, and 303 [88–355] ng/mL for edoxaban. Measured concentrations ranged from 8 to 480 ng/mL for rivaroxaban, from 24 to 194 ng/mL for dabigatran, and from 19 to 481 ng/mL for edoxaban. Dose-specific concentration summaries are presented in Table 3, and individual measurements and concomitant amiodarone use are presented in Table 4.

Two patients had a Cockcroft–Gault creatinine clearance below 50 mL/min while receiving rivaroxaban 20 mg or edoxaban 60 mg. Their measured concentrations were 480 and 461 ng/mL, respectively. No other applicable drug-specific criteria indicated a need for dose reduction among all participants.

For descriptive context, measured values were compared numerically with the published bounds shown in Table 1. Ten of 37 measurements (27.0%) exceeded the published upper bounds of peak concentration distributions: three rivaroxaban measurements exceeded 343 ng/mL and seven edoxaban measurements exceeded 288 ng/mL. Two measurements (5.4%) fell below the published lower bounds of trough distributions: one rivaroxaban measurement was 8 ng/mL and one dabigatran measurement was 24 ng/mL, compared with lower bounds of 12 and 28 ng/mL, respectively.

## 4. Discussion

This study describes randomly timed DOAC plasma concentrations obtained during routine assessment before elective ECV. More than one quarter of measured concentrations exceeded the upper bounds of the expected peak concentration ranges reported in the EHRA Practical Guide. Although this study could not establish the contribution of individual factors, differences in renal function, prescribed dose, concomitant medications, adherence, and the dose-to-sampling interval remain potential explanations for the observed variability in DOAC concentrations [13,19,20].

Two patients with creatinine clearance below 50 mL/min were receiving rivaroxaban 20 mg or edoxaban 60 mg, with measured concentrations of 480 and 461 ng/mL, respectively. These observations highlight the importance of periodically reassessing prescribed DOAC doses against patients’ current clinical characteristics, using the criteria applicable to the individual DOAC, as renal function, body weight, and concomitant medications may change over time [13].

Concentrations outside the reference bounds do not, by themselves, establish excessive or insufficient anticoagulation, because these population-derived ranges are not validated therapeutic thresholds and sampling was not standardized [19,20,21]. No complications were recorded during ECV or before discharge, but no post-discharge follow-up was conducted. Consequently, the absence of recorded complications cannot establish the clinical safety of the observed concentrations.

Patient-related factors may contribute to variation in DOAC plasma concentrations. Renal impairment may reduce drug clearance, while hepatic dysfunction and extremes of body weight may also influence pharmacokinetics, with effects differing between individual DOACs [10,13]. Concomitant medications may further modify concentrations through interactions involving P-glycoprotein and, for some DOACs, cytochrome P450 enzymes. Amiodarone is particularly relevant in the cardioversion setting because of its established role in rhythm control, including in patients with structural heart disease or reduced left ventricular systolic function, where other treatment options may be limited [22]. Its potential to interact with DOACs provides a rationale for documenting its use alongside concentration measurements, although previous studies have reported varying findings regarding its effects on DOAC exposure and bleeding risk [12,23,24,25].

Drug-specific assays, including calibrated anti-FXa and anti-FIIa assays, allow quantitative assessment of DOAC plasma concentrations. However, the clinical role of routine DOAC concentration measurement remains limited both in Latvia and internationally. Despite the availability of these measurements, their clinical application remains limited compared with vitamin K antagonists, for which INR monitoring is supported by defined therapeutic targets. The timing of blood sampling relative to the last dose is essential for interpreting DOAC concentrations before cardioversion. Validated concentration thresholds for routine dose adjustment or management before elective ECV have not been established [19,20,26]. European and U.S. guidelines therefore do not recommend routine measurement of DOAC plasma concentrations, reserving it for selected situations such as suspected non-adherence, acute bleeding, or urgent invasive procedures [13,22,27,28].

Another important factor influencing DOAC plasma concentrations is patient adherence to therapy. It has been demonstrated that patients do not always disclose non-use of anticoagulant medication, and fear of bleeding represents a reported reason for inadequate adherence to treatment [29]. Although adherence was assessed through patient report, individual dose omissions were not systematically documented, and their contribution to low concentrations could not be determined. Importantly, ECV is one of the few procedures for which uninterrupted anticoagulant therapy is mandatory, as it reduces the risk of thromboembolic complications during and after the procedure. Clear patient counselling should address the need to continue anticoagulant therapy before and after cardioversion [3,6].

The principal limitation of this study was the lack of standardized timing between the last DOAC dose and blood sampling. Exact dose-to-sampling intervals were not systematically recorded, limiting pharmacokinetic interpretation. Measurements were therefore described as randomly timed concentrations, with expected ranges used for descriptive context. Additional limitations include the small sample size, the short recruitment period, and the absence of prospective clinical follow-up. Repeated sampling and additional follow-up were not feasible within the available resources and the scope of patient consent. Nevertheless, the study provides individual DOAC concentration measurements obtained during routine pre-ECV care, offering a basis for larger prospective studies with standardized sampling and clinical follow-up.

Despite the existence of interactions between DOACs and other medications, routine therapeutic drug monitoring is generally not required [13,30,31]. Nevertheless, data on the clinical relevance of measuring DOAC plasma concentrations prior to ECV remain limited. Larger prospective studies with standardized sampling and clinical follow-up are needed to investigate the determinants of DOAC concentrations and their potential clinical relevance in this setting.

## 5. Conclusions

More than one quarter of randomly timed DOAC concentrations measured before elective ECV exceeded the upper bounds of expected peak concentration distributions. The observed discrepancies between prescribed doses and current renal function highlight the clinical importance of periodically reassessing dose appropriateness as patient characteristics and concomitant treatments change, according to the criteria specific to each DOAC. Larger prospective studies with standardized sampling and clinical follow-up are needed to clarify the clinical significance of these findings.

## Figures and Tables

**Table 1:** Peak and trough plasma concentration ranges of DOACs in patients treated for AF *.

	Rivaroxaban	Edoxaban	Dabigatran
Peak levels *	178–343	101–288	52–383
Trough levels *	12–137	12–43	28–215

* [ng/mL] the 5th–95th percentiles for rivaroxaban and edoxaban and the 10th–90th percentiles for dabigatran. These population-derived ranges are not validated therapeutic thresholds.

**Table 2:** Demographic Data and Health Status Characteristics of Study Participants.

Patient Characteristics	Total (n = 37)	Gender	
		Female (n = 13)	Male (n = 24)
DOAC			
Rivaroxaban, n (%)			
15 mg	1 (2.7)	0	1 (4.2)
20 mg	17 (45.9)	8 (61.5)	9 (37.5)
Dabigatran, n (%)			
110 mg	3 (8.1)	1 (7.7)	2 (8.3)
150 mg	3 (8.1)	0	3 (12.5)
Edoxaban, n (%)			
60 mg	13 (35.1)	4 (30.8)	9 (37.5)
Age, years, mean (±SD)	62.8 (16.7)	65.0 (21.8)	61.6 (13.5)
Weight, kg, mean (±SD)	85.6 (28.7)	70.3 (40.4)	94.0 (15.2)
CHA2DS2-VA score			
0, n (%)	5 (13.5)	1 (7.7)	4 (16.7)
1, n (%)	6 (16.2)	3 (23.1)	3 (12.5)
≥2, n (%)	25 (67.6)	8 (61.5)	17 (70.8)
HAS-BLED score			
0, n (%)	17 (45.9)	6 (46.2)	11 (45.8)
1–2, n (%)	18 (48.6)	6 (46.2)	12 (50.0)
≥3, n (%)	2 (5.4)	1 (7.7)	1 (4.2)
Stroke (Ischemic), n (%)	1 (2.7)	1 (7.7)	0
Transient ischemic attack, n (%)	1 (2.7)	0	1 (4.2)
Bleeding Events			
Requiring healthcare professional, n (%)	0	0	0
Managed at home, n (%)	4 (10.8)	0	4 (16.7)
CrCl (Cockcroft-Gault) *, mL/min (±SD)	86.0 (38.4)	75.5 (35.4)	91.7 (39.4)
ALT, U/L (±SD)	38.1 (26.6)	34.3 (17.6)	40.0 (30.4)
LDL-C, mmol/L (±SD)	2.2 (1.0)	2.5 (0.9)	2.1 (1.0)
D-dimer, mg/L (±SD)	0.4 (0.3)	0.5 (0.4)	0.3 (0.3)

* Actual body weight was used in the Cockcroft–Gault equation to estimate creatinine clearance. CrCl—creatinine clearance; ALT—alanine aminotransferase; LDL-C—low-density lipoprotein cholesterol.

**Table 3:** Summary of randomly timed DOAC plasma concentrations by prescribed dose.

DOAC Used	DOAC Dose (Number of Patients, n)	Median Concentration, ng/mL [Q1–Q3] *	Minimum–Maximum Concentration, ng/mL
Rivaroxaban	15 mg (n = 1)	164 **	164 **
	20 mg (n = 17)	180 [100–248]	8–480
Dabigatran	110 mg (n = 3)	91 [57–108]	24–126
	150 mg (n = 3)	193 [166–193]	140–194
Edoxaban	60 mg (n = 13)	303 [88–355]	19–481

* Q1 and Q3 denote the first and third quartiles; ** For the subgroup containing one patient, the individual concentration is reported without quartiles or a range.

**Table 4:** Individual randomly timed DOAC plasma concentrations and concomitant amiodarone use.

Patient No.	DOAC	DOAC Dose	DOAC Concentration, ng/mL	Comparison with Reference Bounds *	Comment
1	Rivaroxaban	15 mg	164	–	–
2		20 mg	8	Below lower bound	
3			29	–	Used amiodarone
4			52	–	–
5			98	–	Used amiodarone
6			100	–	Used amiodarone
7			115	–	Used amiodarone
8			143	–	Used amiodarone
9			172	–	–
10			180	–	–
11			183	–	Used amiodarone
12			194	–	–
13			242	–	Used amiodarone
14			248	–	Used amiodarone
15			268	–	–
16			409	Above upper bound	–
17			450	Above upper bound	Used amiodarone
18			480	Above upper bound	CrCl < 50 mL/min; Used amiodarone
19	Dabigatran	110 mg	24	Below lower bound	–
20			91	–	–
21			126	–	–
22		150 mg	140	–	Used amiodarone
23			193	–	–
24			194	–	Used amiodarone
25	Edoxaban	60 mg	19	–	–
26			21	–	–
27			28	–	–
28			88	–	–
29			160	–	–
30			160	–	–
31			303	Above upper bound	Used amiodarone
32			328	Above upper bound	–
33			353	Above upper bound	Used amiodarone
34			355	Above upper bound	Used amiodarone
35			390	Above upper bound	Used amiodarone
36			461	Above upper bound	CrCl < 50 mL/min
37			481	Above upper bound	Used amiodarone

* “Below lower bound” and “Above upper bound” indicate numerical comparisons with the lower bounds of trough distributions and upper bounds of peak distributions shown in Table 1, respectively.

## Data Availability

The original contributions presented in this study are included in the article. Further inquiries can be directed to the corresponding author.

## Abbreviations

The following abbreviations are used in this manuscript:

ECV	Electrical cardioversion
AF	Atrial fibrillation
DOACs	Direct oral anticoagulants
ESC	European Society of Cardiology
P-gp	P-glycoprotein
PSCUH	Pauls Stradiņš Clinical University Hospital
ALT	Alanine aminotransferase
LDL-C	Low-density lipoprotein cholesterol
CrCl	Creatinine clearance
